# A Rare Case of Achalasia Sigmoid Esophagus Obstructed by Food Bolus

**DOI:** 10.7759/cureus.45567

**Published:** 2023-09-19

**Authors:** Charles Vallejo, Yousra Gheit, Talwinder K Nagi, Zoilo K Suarez, Muhammad A Haider

**Affiliations:** 1 Internal Medicine, Florida Atlantic University, Boca Raton, USA

**Keywords:** esophago-gastro-duodenoscopy, food impaction, sigmoid esophagus, severe achalasia, esophageal achalasia

## Abstract

Achalasia is a primary esophageal motility disorder that involves a failure of the lower esophageal sphincter to relax in response to swallowing. Specifically, the lower esophageal sphincter becomes hypertensive, and there is an absence of peristalsis in the esophagus. The pathophysiology is thought to be due to a loss of inhibitory nerve function from an autoimmune attack that targets the esophageal myenteric nerves. As a result, these abnormalities lead to a functional obstruction at the gastroesophageal junction. In severe cases, achalasia may present as a “sigmoid esophagus,” a term used to describe the dilation and distortion of the cervical esophagus. In this case report, we discuss a patient with a known history of achalasia who presented with extra-esophageal symptoms including respiratory distress and tracheal compression from an esophagus dilated with a food impaction. She was found to have a sigmoid esophagus and required direct endoscopy and removal of the food bolus. We will review the pathogenesis of achalasia as well as medical and surgical approaches to treating severe achalasia as presented through other case reports.

## Introduction

Achalasia is an esophageal smooth muscle motility disorder that occurs when the lower esophageal sphincter fails to relax. Specifically, it is thought that achalasia occurs from the degeneration of the myenteric plexus and vagus nerve fibers in the lower esophageal sphincter. Due to this, there is a loss of inhibitory neurons containing vasoactive intestinal peptide and nitric oxide synthase [1]. As a result, the lower esophageal sphincter becomes non-relaxed and hypertensive. Subsequently, a functional obstruction develops at the gastroesophageal junction. Notably, there is also a marked absence of peristalsis in the esophagus. Currently, the etiology and mechanism of the degeneration are not fully understood, although there are theories suggesting that autoimmune phenomena, viral infections such as Trypanosoma cruzi/Chagas disease, neurodegenerative disorders, and eosinophilic gastroenteritis may play an important role [1].

In general, achalasia is rare with a prevalence of about 10 per 100,000 people. Patients usually present with dysphagia and regurgitation of both solids and liquids at presentation. Some patients also present with chest pain, aspiration, heartburn, and weight loss from difficulty in eating. The initial test for evaluation is a barium esophagogram which may demonstrate a “bird’s beak” appearance with dilatation of the proximal esophagus. Subsequently, an esophagogastroduodenoscopy (EGD) is recommended to exclude premalignant or malignant lesions [1]. Esophageal manometry may determine the disease’s diagnosis with high certainty, even during an early stage [2].

We present a case of severe achalasia leading to extra-esophageal symptoms including respiratory distress and tracheal compression from an esophagus dilated with a food impaction. Specifically, the patient presented with a “bullfrog neck” appearance from severe dilation and distortion of the cervical esophagus and was termed to have a “sigmoid esophagus” on EGD secondary to the dilatation.

## Case presentation

**Figure 1:**
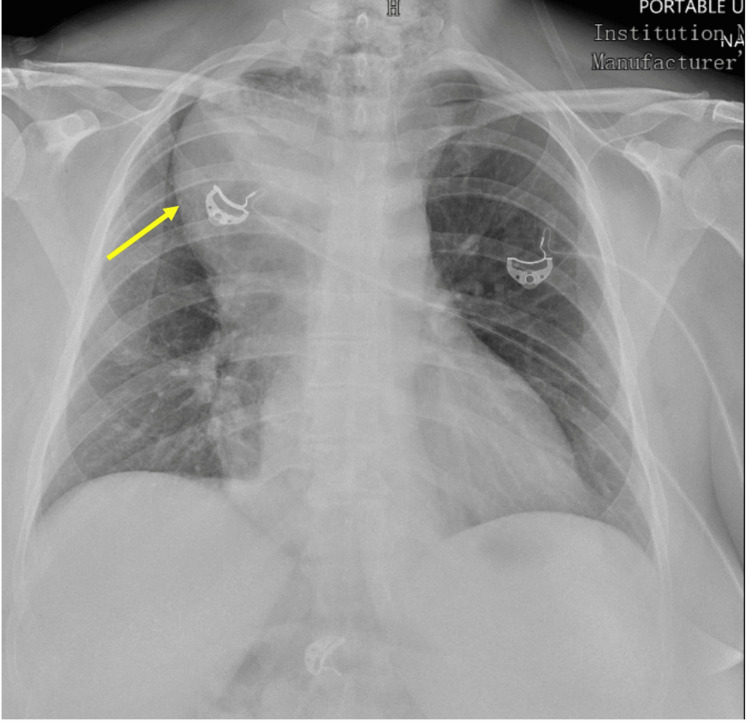
Chest X-ray on admission depicting broadening of the mediastinum

The patient is a 50-year-old female with no past medical history, who presented to the emergency room in respiratory distress immediately after having a breakfast sandwich. On arrival, her vital signs were significant for blood pressure 148/96 mmHg, heart rate of 140 beats per minute, and respiratory rate of 44 breaths per minute. The patient endorsed dysphagia to both solids and liquids which was progressively increasing for the past 12 years. She admitted to seeing multiple outpatient gastroenterologists who had diagnosed her with achalasia and recommended that she undergo an esophageal dilation, but she was lost to follow-up. Chest X-ray on admission was notable for broadening of the mediastinum as shown in Figure 1. A soft tissue X-ray of her neck revealed a mediastinal mass obstructing the esophagus. Therefore, both a gastroenterologist and an otorhinolaryngologist (ENT) were consulted for esophageal food disimpaction. The patient was taken to the operating room for direct endoscopy and removal of the food bolus. Full pieces of undigested food bolus were removed during the procedure. She remained nil per orally (NPO) after the procedure.

**Figure 2:**
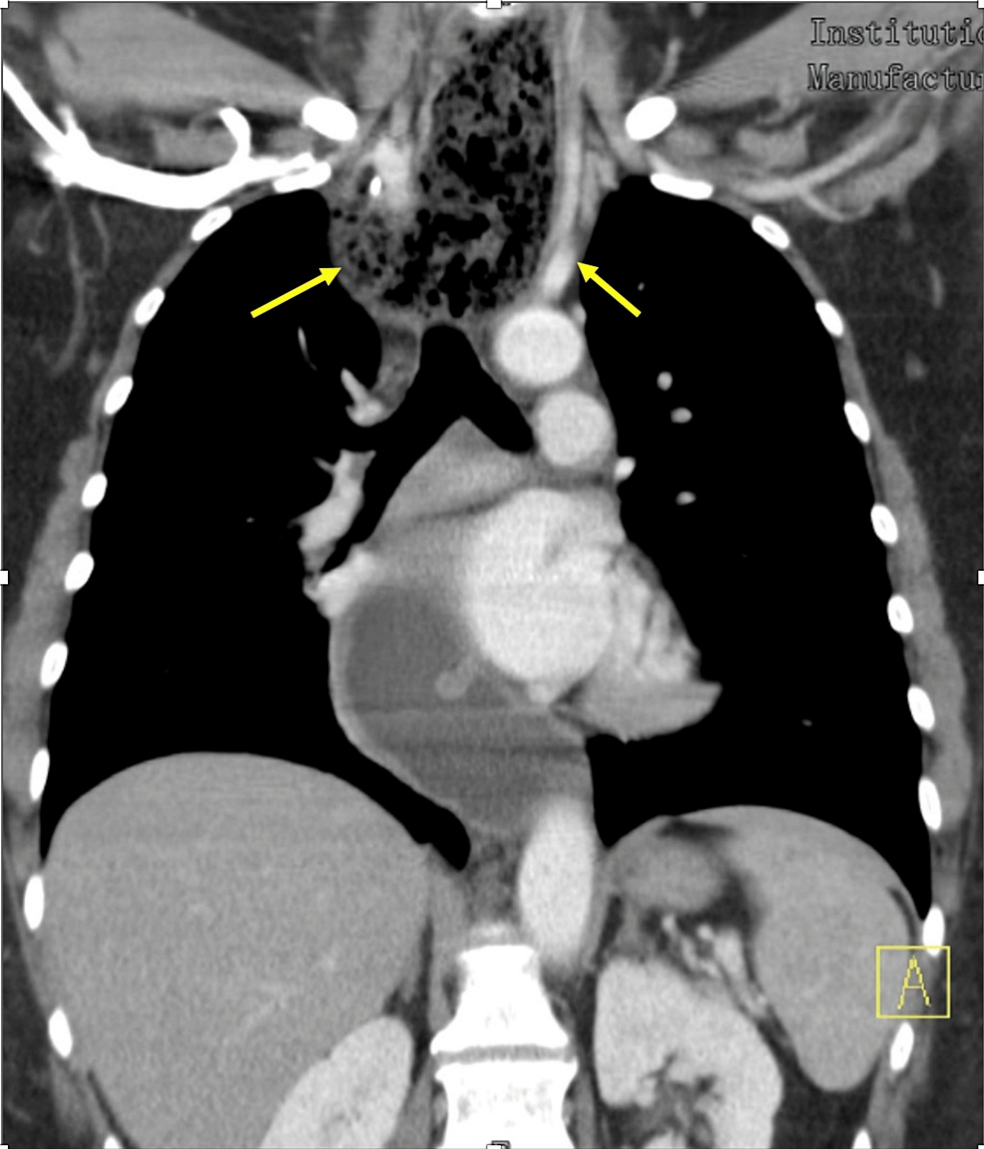
CT scan depicting distended cervical and upper thoracic esophagus suggesting a distal obstruction

The following day, a neck CT scan was done and revealed a distended cervical and upper thoracic esophagus suggesting a distal obstruction, as shown in Figure 2. There was concern for a distal mass versus stricture. On day two of her admission, she underwent an EGD. However, upon entering the esophagus, significant liquid and food residue were seen, and the procedure was aborted. She underwent repeat EGD with intubation two days later while remaining NPO. The repeat EGD showed a severely dilated esophagus, consistent with a “sigmoid esophagus," and the esophageal mucosa appeared inflamed. In an attempt to provide the patient with symptomatic relief, Botox was injected into the lower esophageal sphincter (LES). This is because the esophageal narrowing appeared to mainly be in the lower one-third of the esophagus and the LES, while the food bolus was obstructing proximally as seen during the EGD. Additionally, a balloon dilation was performed. Because these interventions were not successful, she eventually required a jejunostomy feeding tube (J-tube) placement.

It should be noted that a Heller myotomy was recommended by several outpatient gastroenterologists the patient had previously seen over the past 12 years, but the patient did not adhere to these recommendations or follow-up. During her inpatient stay, the consulting gastroenterologists suggested that neither a Heller myotomy procedure nor a peroral endoscopic myotomy (POEM) would provide substantial benefit in the setting of this advanced stage achalasia. The patient also did not wish to further investigate the etiology of her achalasia in an inpatient or outpatient setting, and therefore, it remained unknown in this case. Ultimately, she was discharged from the hospital on her seventh day of admission with a J-tube for feeding.

## Discussion

Through the literature review, only a few cases of advanced achalasia have been described. Advanced achalasia may present similarly to our patient discussed here, with respiratory distress and progressive dysphagia. The extra-esophageal manifestations of achalasia should not be undermined as tracheal compression and respiratory distress may result, such as presented in our case. Likewise, one case report presents achalasia with mega-esophagus and tracheal compression in a 23-year-old male patient. The patient complained of weight loss, dysphagia, regurgitations, and respiratory ailments. His labs were unrevealing for any abnormalities, but a CT scan showed a massive extension of the esophagus from the cardia and up to the cervical vertebral body 7. There was also mediastinal widening and narrowing of the trachea. He underwent a laparoscopic Heller myotomy. Three and a half years later, the patient reported resolution of dysphagia, regurgitation, and respiratory ailments [3]. The authors suggest that other respiratory symptoms associated with achalasia include pneumonia, bronchiectasis, and aspiration. Only a few publications have reported neck swelling or “bullfrog neck” such as the patient presented in our case. When severe, this may lead to progressive dyspnea and acute total airway collapse [3].

Similar to our patient, one case report presents a 37-year-old male with no past significant medical history who experienced nine months of weight loss, vomiting, and dysphagia. A CT scan was performed and was compared to a CT scan performed seven months prior. There was an increased dilation of the proximal esophagus with debris. There was also wall thickening of the esophagus, gastroesophageal junction, and stomach. The patient was initially treated with pantoprazole but failed medical treatment and, therefore, underwent endoscopic Botox injection. Additionally, the patient was deemed a good surgical candidate and underwent laparoscopic Heller myotomy. Subsequently, he reported immediate improvement to his dysphagia, was discharged with a Jackson-Pratt drain two days later, and followed up two weeks later. The authors propose that as seen in our case, advanced achalasia may lead to esophageal dilatation with food stasis and a sigmoid-like appearance [2]. The procedure of choice is laparoscopic Heller myotomy, but other options such as pneumatic dilation and POEM are sometimes also utilized.

The importance of surgical intervention for advanced achalasia with sigmoid esophagus is discussed in several case reports. A case report of a 34-year-old female describes the patient’s clinical presentation with worsening dysphagia and malnourishment secondary to advanced achalasia. An EGD was unsuccessful due to the presence of undigested food in the lower esophagus, but a barium study revealed a bird’s beak appearance. An esophageal manometry confirmed the diagnosis and was consistent with poor esophageal function. The patient was treated with myotomy and Dor fundoplication which seemed to be highly effective [4].

## Conclusions

Advanced achalasia may present with typical symptoms such as dysphagia and regurgitation with both solids and liquids at presentation. However, it may additionally accompany extra-esophageal symptoms such as respiratory distress in the setting of tracheal compression as presented in our case. Symptoms may not always improve with Botox injection through endoscopic intervention into the lower esophageal sphincter, and therefore, a J-tube placement may be necessary.
